# Radiomics in fetal brain MRI: a narrative review

**DOI:** 10.1186/s41747-026-00697-z

**Published:** 2026-03-16

**Authors:** Francesco Pacchiano, Mario Tortora, Valentina Bordin, Francesca Gentile, Mario Cirillo, Fabio Tortora, Ferdinando Caranci, Lorenzo Ugga

**Affiliations:** 1 University of Naples “Luigi Vanvitelli”, Naples, Italy; 2 University of Naples “Federico II”, Naples, Italy; 3 Politecnico di Milano (Polytechnic University of Milan), Milan, Italy

**Keywords:** Brain, Fetus, Magnetic resonance imaging, Prenatal care, Radiomics

## Abstract

**Abstract:**

Fetal MRI has emerged as a crucial supplement to prenatal ultrasonography in the evaluation of the developing brain and in identifying congenital defects and minor developmental malformations. While fetal brain MRI interpretation has always depended on visual examination of signal properties and morphology, images can provide quantitative information that could be missed or hidden from the human eye. Radiomics allows for characterizing tissue characteristics and heterogeneity by extracting quantitative information from imaging data. In this narrative review, after summarizing the technical foundations of fetal MRI radiomics (acquisition, preprocessing, segmentation, feature extraction and types, machine learning models, feature reproducibility and quality), we consider the following major clinical applications: brain development assessment and phenotyping; Chiari II malformation and brain edema phenotype; isolated ventriculomegaly and prediction of its persistence; and prognosis and neurodevelopmental outcome prediction. MRI radiomics presents a promising technique to improve the assessment of the fetal brain. Larger multicenter studies with standardized protocols are essential to improve generalizability and reduce variability. Combining radiomics with deep learning could enhance performance and interpretability, while biological validation, linking features to known tissue properties, will help confirm clinical relevance.

**Relevance statement:**

Despite its early stage, MRI radiomics offers a new, data-driven lens to evaluate fetal brain development. By revealing subtle imaging patterns not visible to the eye, it may eventually support more accurate diagnosis, risk stratification, and personalized care.

**Key Points:**

- Fetal MRI adds value beyond ultrasound in the prenatal setting.
- Radiomics reveals hidden imaging features.
- Radiomics enhances diagnosis and prognosis in fetal brain assessment.
- Large multicenter studies are needed.

**Graphical Abstract:**

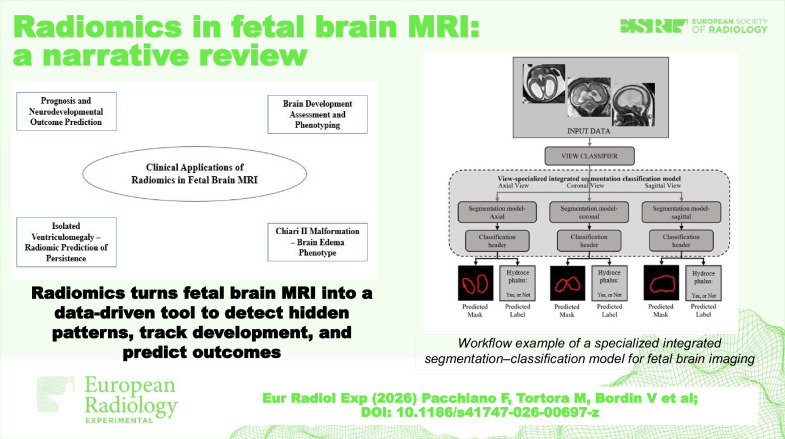

## Background

Fetal brain development is a dynamic and sensitive process that can be affected by a range of genetic and environmental factors. Accurate assessment during pregnancy is crucial for early diagnosis of brain abnormalities, counseling, and planning postnatal care. While prenatal ultrasonography remains the first-line modality for fetal neuroimaging, magnetic resonance imaging (MRI) has emerged as a powerful complementary tool, offering superior contrast resolution and a broader field of view [1]. Fetal brain MRI, however, presents unique technical challenges: unpredictable fetal motion, small and evolving anatomy, variable tissue contrast, and limitations in acquisition protocols. To mitigate motion artifacts, fast T2-weighted two-dimensional (2D) sequences are typically used, often requiring postprocessing techniques to reconstruct three-dimensional (3D) volumes. Interpretation of these images has traditionally been qualitative, relying on expert visual assessment.

Radiomics is an emerging technique that objectively quantifies tissue features and heterogeneity by extracting large-scale data from medical images. By converting images into mineable datasets, it can uncover subtle patterns of texture, shape, and intensity that may reflect microstructural changes or hidden disease, often invisible to standard visual inspection [1, 2]. Radiomics analysis of the fetal brain involves a multistep pipeline: image acquisition, preprocessing, region-of-interest segmentation, feature extraction, and model building, typically *via* machine learning (ML) [3–5].

Each step presents unique challenges in the fetal MRI setting. In fetal MRI, motion-corrected volumetric reconstructions or multiplanar image series are first obtained from fast sequences (commonly single-shot T2-weighted images). The target region (such as the whole brain or a specific structure) must then be delineated, either manually or with automated segmentation algorithms. From these defined regions of interest (ROIs), many mathematical features can be computed to quantify the tissue’s intensity distribution, texture patterns, and shape geometry. These features are the radiomic signature of the ROI and can be mined for associations with clinical labels or outcomes using statistical or machine learning models [1, 2].

## Technical foundations of fetal brain radiomics

### Image acquisition and preprocessing

Fetal brain MRI is typically performed without maternal or fetal sedation, using rapid sequences (T2-weighted half-Fourier acquisition single-shot turbo spin-echo (HASTE) or fast spin-echo (FSE) sequences, balanced steady-state free precession (SSFP), etc.) to reduce motion artifacts [6, 7]. These sequences yield stacks of 2D slices, often in multiple planes (axial, sagittal, coronal). To facilitate radiomics, which ideally analyzes consistent 3D volumes, motion correction and super-resolution reconstruction algorithms (*e.g*., slice-to-volume registration) can be applied to create an isotropic 3D image of the fetal brain [1]. Standardizing voxel size and intensity is important because radiomic features are sensitive to these factors [8]. Many studies normalize image intensity and resample to a fixed voxel spacing prior to feature extraction, for example, Shi et al normalized fetal T2 images and resampled to 2 × 2 mm in-plane resolution in their radiomics analysis of the brain [8]. Such preprocessing ensures that features (like texture frequencies) reflect true tissue differences rather than scanner or sequence parameters. Unlike CT, MRI voxel intensities are not quantitative in absolute terms as they depend on variables such as sequence parameters, field strength, and tissue relaxation properties [9]. To mitigate this variation, preprocessing includes intensity normalization (for example, with z-score) and bias field correction, which removes the gradual shading in MRI images caused by uneven signal, ensuring that similar tissue looks equally bright across the image, such as the widely used N4 bias field correction algorithm [10, 11]. In addition, resampling to isotropic voxels is recommended to avoid slice thickness biasing the texture features [2]. Compared to radiomics in adult subjects, fetal brain MRI presents unique challenges such as fetal motion, dynamic anatomy, and variable tissue contrast, necessitating rapid 2D imaging and advanced preprocessing. These factors require customized normalization and harmonization to enable consistent and reliable radiomic feature extraction.

### Image segmentation

A critical prerequisite for fetal brain radiomics is reliable segmentation of the whole brain or subregions, challenging due to motion, changing anatomy with gestation, and lower image contrast compared to postnatal MRI [12]. Early studies have commonly relied on expert manual segmentation of a 2D slice or 3D volume [13]. Although manual ROIs ensure anatomical accuracy, they are time-consuming and potentially variable between raters. Recent technical developments include automated fetal brain segmentation networks often deep learning based that can generate 3D masks of brain tissues from motion-corrected MRI volumes. Several approaches, including U-Net variants and transformer-based models, were recently presented at the Fetal Tissue Annotation and Segmentation Challenge (FeTa 2022), demonstrating strong performance and good generalization across institutions and gestational ages [12]. In parallel, Mazher et al (2022) introduced a multi-view deep learning model, the IRMMnet, to segment fetal brain tissues, which then enabled radiomics feature extraction from standardized regions across cases [13]. Such approaches aim to reduce operator dependence and allow radiomics to be applied at scale.

### Feature extraction and types

Once an ROI is defined, quantitative features describing the voxel intensity statistics and spatial patterns are computed [14]. Common radiomic feature classes include: (1) first-order intensity statistics (*e.g*., mean, variance, skewness of voxel intensities); (2) shape descriptors (*e.g*., volume, surface area, compactness of the ROI if a 3D volume is segmented); and (3) texture features derived from matrices like the gray-level co-occurrence matrix, gray-level run-length matrix, gray-level size zone matrix, etc., which capture patterns of pixel intensity correlations and heterogeneity [15]. In fetal brain MRI, texture features computed on T2-weighted images can reflect subtle differences in microstructure or maturation of tissues. Many pipelines also apply filters (such as wavelet decompositions or Laplacian-of-Gaussian filters) to the images and extract features from these transformed images, expanding the feature [4, 16]. Feature selection is usually performed to reduce dimensionality: algorithms identify the most informative features that correlate with the outcome of interest, discarding redundant or low-variance features. Common selection methods include correlation-based filtering or sparsity-promoting models such as the Least Absolute Shrinkage and Selection Operator (LASSO) to choose a subset of features that best discriminate against classes [13]. For instance, in a gestational age prediction task, features such as entropy, energy, and shape measures (elongation, sphericity, etc.) were selected from an initial set of 108 features as the best predictors of brain age [13].

### Machine learning models

The final step uses the selected radiomic features as inputs to a predictive model. Given relatively small sample sizes in fetal studies, simple machine learning classifiers/regressors are typically employed rather than very deep networks. Examples include logistic regression, support vector machines, random forests, and gradient-boosted trees [13]. These models are trained (with cross-validation or hold-out validation) to either classify categories (*e.g*., diseased *versus* normal) or predict continuous outcomes (*e.g*., gestational age, future measurements). In the fetal brain radiomics literature, models have been built to distinguish different fetal conditions or to predict postnatal results. Chen et al [17] used multivariable logistic regression with cross-validation on radiomics features to predict which fetuses with ventriculomegaly would have persistent postnatal ventricular enlargement. Mazher et al [13] tested several regression algorithms (random forest, regression trees, etc.) to map radiomic features to gestational age, finding that random forest performed best. Because overfitting is a concern with high-dimensional radiomic data, rigorous validation is crucial. Many studies report performance on a separate validation set or *via* cross-validation using the area under the receiver operating characteristic curve (AUROC) or root mean square error for continuous outcomes as metrics of generalization.

### Feature reproducibility and radiomics quality considerations

An important technical consideration is the reproducibility of radiomic features. Fetal MRI adds complexity here because fetal motion or slice positioning can vary between scans. A recent reproducibility study by Watzenboeck et al examined radiomic features in repeated fetal MRI acquisitions (though of the lung) and found that 3D volumetric radiomics features had significantly higher reproducibility than features derived from single 2D slices [18, 19]. While that study focused on lungs, the principle likely could be extended to the brain: whenever possible, using the whole 3D brain ROI yields more robust features, whereas analysis of one cross-sectional slice may be more noise-prone and less representative. Additionally, that work highlighted that only a subset of features achieved excellent test–retest repeatability, mostly first-order intensity features [18]. This underscores the need to validate fetal radiomics features under varying conditions. Standardization initiatives, such as the Image Biomarker Standardization Initiative (IBSI), and adherence to the Radiomics Quality Score criteria (which emphasize repeatability, multiple-center data, and external validation) are recommended to bolster confidence in fetal radiomics research [3, 18]. So far, most fetal brain radiomics studies have been single-center and retrospective, and reproducibility assessments remain sparse. As the field progresses, greater emphasis on harmonizing MRI protocols (*e.g*., 1.5-T *versus* 3-T field strength effects) and on sharing open fetal MRI datasets will help ensure that radiomic biomarkers are not site-specific quirks but rather truly generalizable findings, similar to what was proposed for METRICS (METhodological RadiomICs Score), a consensus-based scoring tool developed by international experts to assess the methodological quality of radiomics studies [20]. Table 1 summarizes the papers that focused on the technical and analytical methodologies in fetal brain radiomics.

**Table 1:** Studies focusing on the technical and analytical methodologies in fetal brain radiomics

First author, year [reference number]	Objective	Main finding
Mazher, 2022 [13]	Brain segmentation and gestational age estimation	Radiomic features predicted gestational age with ~1.4-week error
Payette, 2024 [12]	Automated segmentation challenge (FeTa 2022)	Good performance across gestational ages

## Clinical applications of radiomics in fetal brain MRI

### Brain development assessment and phenotyping

**Fig. 1:**
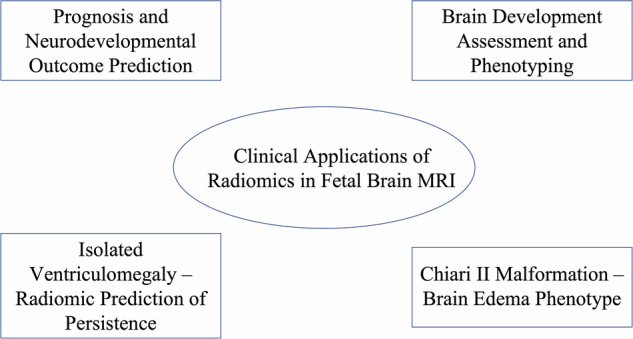
Overview of the main clinical applications of radiomics in fetal brain MRI

One major application of radiomics is the quantitative assessment of fetal brain development. The growth and maturation of the brain in utero involves microstructural changes (*e.g*., increasing complexity of gyration, myelination, and organization of developing gray/white matter) that may not be fully captured by standard biometric measurements. Radiomics can potentially serve as an advanced imaging biomarker of gestational brain maturity or detect subtle deviations in development due to pathological influences. Figure 1 recaps the main clinical use of radiomics in fetal brain imaging.

Early work by Sanz-Cortés et al [21] indicated that MRI texture features could distinguish small-for-gestational-age fetuses from those with appropriate growth. In their study, term fetuses with growth restriction showed altered texture in several brain regions compared to normal fetuses, allowing classification with around 91–99% accuracy.

The study of Mazher et al [13] tackled gestational age estimation from fetal brain MRI as a proxy for brain development. The authors combined deep learning segmentation of the brain with radiomic feature extraction to predict the “brain age” of fetuses ranging from 20 to 33 weeks of gestation [13]. In their approach, over a hundred radiomic features (including shape descriptors like axis lengths and surface area, and texture measures like entropy and contrast) were computed from each fetus’s MRI. Using a random forest regressor on selected features, the model achieved an error of approximately 1.4 weeks in estimating gestational age from the MRI alone [13]. The result suggests that radiomic features of the fetal brain change in a predictable way as gestation progresses, essentially quantifying normal brain maturation. Such an approach could be useful in clinical scenarios where the gestational age is uncertain or to detect when a fetus’s brain appears developmentally “younger” or “older” than expected, especially in a context with limited expertise to review the images. It is worth noting that this was a single-center study with a relatively small sample size (80 fetuses) and, primarily, a technical feasibility demonstration.

A recent study in neonates born to gestational diabetic mothers used MRI texture analysis to identify microstructural differences in the newborn brain [22]. They found that certain texture features on neonatal MRI were able to distinguish babies exposed to maternal diabetes from controls with high accuracy (AUROC: 0.98). The authors concluded that gestational diabetes influences brain development detectable from fetal life to the neonatal period [22]. By extension, one can anticipate that radiomics applied directly on fetal MRI might detect these changes even before birth. These results offer new possibilities for detecting subtle changes and identifying which newborns could be more affected than others.

Notably, the current literature indicates that radiomic features correlate strongly with gestational maturation and can detect growth-related microstructural alterations. However, normative reference ranges of radiomic features across gestation are not yet established, and external validation of gestational age prediction models (perhaps on multicenter data or by comparison to ultrasound-based gestational age) is needed. Still, these initial studies demonstrate the feasibility of radiomics as a quantitative measure of fetal brain development.

### Chiari II malformation and brain edema phenotype

In the case of Chiari II malformation, fetal MRI is crucial to assess hindbrain herniation and plan possible in utero repair surgery.

Shi et al [8] used radiomics to investigate prenatal brain edema, which can be a prenatal MRI finding in Chiari II. They retrospectively studied 91 fetuses with Chiari II, of which about half had MRI signs of supratentorial brain edema. The authors used a manual 2D segmentation of the brain parenchyma on a mid-axial slice to extract 783 radiomics features (after normalization and filtering) from each fetus’s T2 image. A gradient-boosted tree model (XGBoost) was trained to classify cases with edema *versus* those without. The radiomics model achieved a cross-validated AUROC of 0.81 in differentiating edema-positive *versus* edema-negative Chiari II fetuses [8]. The ten most important features were predominantly texture metrics (gray-level co-occurrence matrix and related matrices) reflecting gray-level non-uniformity and run-length patterns (likely to capture the blurring of normal cortical texture in edema) [8]. This performance is notable when considering that standard biometric measures (like ventricle size or posterior fossa measures) are often similar between those cases. Radiomics provided a new way to quantify the degree of microstructural disturbance. Moreover, when the model was used to separate any Chiari II brain (edematous or not) from normal fetuses, the AUROC was extremely high (0.98), indicating radiomic features easily detect the presence of Chiari-related changes *versus* a healthy brain. Clinically, the presence of diffuse brain edema in Chiari II was correlated with worse motor outcomes after birth and could influence the decision to perform fetal surgery. The radiomics approach by Shi et al [8] provides an objective way to identify this severe phenotype in utero. One limitation, however, is that they used only a single 2D slice ROI, which may not capture the full extent of pathology; this choice was likely to avoid errors from 3D reconstruction. Nonetheless, this work illustrates radiomics’ potential in anomaly subclassification, in this case, distinguishing a high-risk subgroup (with edema) from a lower-risk subgroup of the same malformation based on imaging features.

### Isolated ventriculomegaly and prediction of its persistence

Fetal ventriculomegaly, defined as enlargement of the lateral ventricles (typically > 10 mm atrial diameter), is a common finding with a wide spectrum of outcomes. Depending on the case and the cause, some may remain stable, and others may progress to hydrocephalus.

**Fig. 2:**
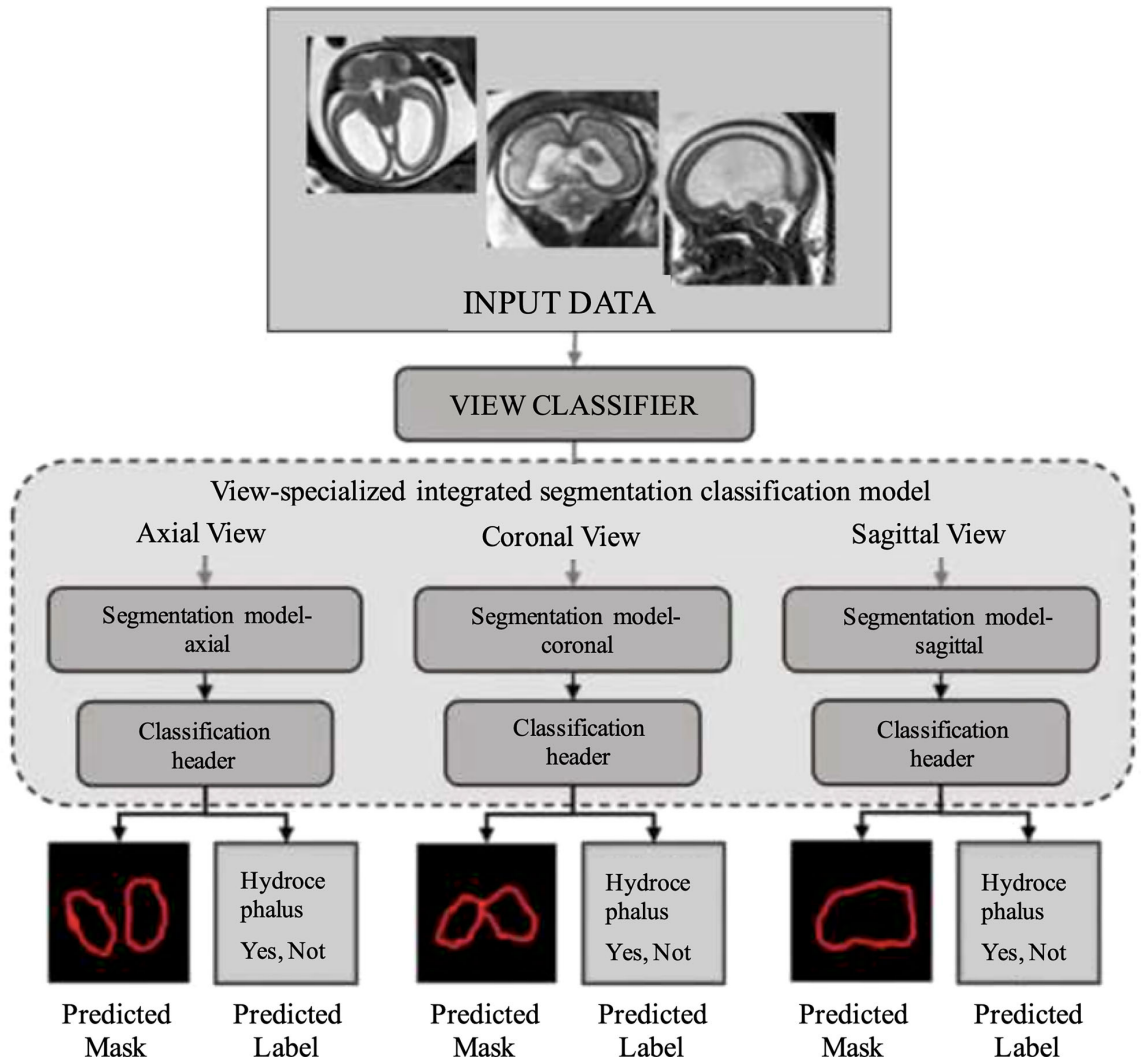
Workflow example of a specialized integrated segmentation–classification model for fetal brain imaging. Input data from multiple orientations (axial, coronal, sagittal) are first routed through a view classifier, which directs images to view-specific segmentation models. Each segmentation branch generates predicted masks of the ventricular system, followed by a classification header that determines the presence or absence of hydrocephalus. This is an example approach highlighting how radiomics and deep learning can be combined to integrate segmentation and diagnostic classification in fetal neuroimaging, supporting diagnosis

Chen et al [17] leveraged radiomics to predict which fetuses with apparently isolated mild ventriculomegaly would go on to have persistent or worsening ventricular dilation after birth. They studied 101 fetuses with isolated ventriculomegaly on prenatal MRI, dividing them into resolved and stable groups based on postnatal outcomes. For radiomics analysis, they focused on the brain tissue surrounding the occipital horn of the lateral ventricle (periventricular white matter). Radiomic features from this subcortical region were extracted (likely to include texture that might reflect white matter integrity or gliosis). A logistic regression model was then trained to classify fetuses into the resolved *versus* stable outcome. Notably, the radiomics model achieved an AUROC of ~0.82 in training and ~0.74 on validation for predicting persistent ventriculomegaly. In addition, the radiomic approach showed added value on decision curve analysis, implying it could improve clinical decision-making beyond standard predictors [17]. Furthermore, as a byproduct, the authors found the radiomic features could perfectly distinguish normal fetuses from those with ventriculomegaly (AUROC: 0.99), underscoring that even “isolated” mild ventriculomegaly cases have detectable microstructural brain differences. The drawn conclusion was that microstructural alterations in the periventricular zone can serve as predictive markers for postnatal ventricular outcome [17]. This study adds new elements that could be helpful in predicting which patients may need treatment and which do not. Limitations include the retrospective design and the fact that the MRI sequence used (balanced steady-state free precession) might not be standard in all centers for fetal brain. The ability to predict which fetuses or children will remain compensated and which will go on to develop overt hydrocephalus would be of immense value, particularly since many brain malformations are frequently associated with ventriculomegaly and the eventual onset of hydrocephalus, not easy to predict. Figure 2 shows a schematic of a segmentation–classification pipeline.

### Prognosis and neurodevelopmental outcome prediction

Perhaps the main and most interesting objective of incorporating radiomics into fetal imaging is to improve prognostication, that is, to predict the child’s eventual neurodevelopmental outcomes or need for interventions, above and beyond what current imaging and clinical markers can tell us. This overlaps with anomaly detection, since many anomalies are managed based on anticipated prognosis. The distinction we make here is that outcome-focused studies explicitly link fetal radiomic features to a follow-up outcome measure (postnatal imaging or neurodevelopmental assessments).

The ventriculomegaly study by Chen et al discussed above is one clear example of outcome prediction: the outcome of interest was postnatal ventricular size status, which can be considered deeply related to neurological outcomes, as persistent ventriculomegaly often correlates with developmental delays. Their radiomics model, as noted, provided a risk assessment for needing postnatal intervention [17]. Another indirect example is the Chiari II radiomics study; while it did not itself follow babies postnatally, it identified a fetal imaging phenotype (edema) known to correlate with worse motor outcomes, thereby offering a potential prognostic marker [8]. In both cases, these studies added quantitative elements that could enhance neuroradiologists’ diagnosis.

Another interesting element is the possibility of using fetal radiomics to correlate with the postnatal neurodevelopmental scores. Although no large-scale studies have been reported in this field, a study of preterm neonates at term-equivalent age by Wagner et al [23] found that radiomic features from their brain MRI improved prediction of 2-year neurodevelopmental outcomes beyond clinical variables; this suggests the approach could also be applied in the fetuses’ studies. Moreover, Zheng et al [24] developed an artificial intelligence model using placental MRI to detect preeclampsia and assess its severity. The model combined radiomics and deep learning, achieving a Dice similarity score of 0.918 in differ preeclampsia with fetal growth restriction from normal pregnancies. Lai et al [11] recently conducted a radiomics-based analysis of fetal brain MRI in monochorionic twin pregnancies to explore how subtle microstructural features relate to later neurodevelopmental outcomes. They found that specific texture and shape metrics could distinguish fetuses with favorable from those with moderate developmental trajectories. Furthermore, a logistic regression model incorporating selected radiomic features achieved a high predictive accuracy (AUROC: 0.89), underscoring the potential of prenatal radiomics for early neurodevelopmental risk assessment.

Table 2 summarizes the studies that focus on radiomic applications in the fetal brain. In these articles, we conducted a qualitative assessment of the methodological rigor of the included studies using the METhodological RadiomICs Score (METRICS), a quality scoring tool for radiomics research endorsed by EuSoMII [20]. This evaluation revealed substantial heterogeneity across the analyzed works, with METRICS scores ranging from 37% to 85%, indicating markedly variable methodological quality. Detailed scoring for each individual study is provided in the Supplementary Materials.

**Table 2:** Studies addressing the clinical applications and implications of radiomics in fetal brain MRI

First author, year [reference number]	Objective	Main finding
Sanz-Cortés, 2013 [21]	Small-for-gestational-age *versus* normal fetuses	Radiomics differentiates small-for-gestational-age microstructure
Mazher, 2022 [13]	Gestational age estimation	Achieved highly accurate fetal brain segmentation and gestational age estimation
Shi, 2024 [8]	Chiari II with/without edema	Identifies high-risk edema phenotype
Chen, 2024 [17]	Isolated ventriculomegaly outcome prediction	Predicts persistent postnatal ventriculomegaly
Feng, 2023 [22]	Neonates of diabetic mothers	Detects microstructural impact of maternal diabetes
Lai, 2025 [11]	Monochorionic twin outcomes	Predicts neurodevelopmental risk
Zheng, 2025 [24]	Placental radiomics (PE/fetal growth restriction)	Identify preeclampsia and predict fetal growth restriction
Wagner, 2022 [23]	Very preterm children outcomes	Radiomics and clinical data model predicted neurodevelopment

### Challenges, future directions and conclusions

Fetal brain radiomics holds great potential, but several challenges currently limit its clinical application. Motion artifacts, small sample sizes, and reliance on manual segmentation reduce the reproducibility of results. Many radiomic features also lack clear biological interpretation, and integrating complex models into routine prenatal care remains difficult.

To move the field forward, larger multicenter studies with standardized imaging protocols are essential to improve model generalizability and reduce variability. Technically, combining radiomics with deep learning could enhance both performance and interpretability, while biological validation, linking features to known tissue properties, will help confirm clinical relevance. Addressing these issues will be key to translating radiomics into real-world prenatal decision-making.

Despite its early stage, radiomics offers a new, data-driven lens to evaluate fetal brain development. By revealing subtle imaging patterns not visible to the eye, it may eventually support more accurate diagnosis, risk stratification, and personalized care. With continued research and careful integration into clinical workflows, radiomics could significantly advance the field of fetal neuroimaging.

## Supplementary information

Supplementary materials

## Abbreviations

2D: Two-dimensional
3D: Three-dimensional
AUROC: Area under the receiver operating characteristic curve
METRICS: Methodological radiomics score
MRI: Magnetic resonance imaging
ROI: Region of interest
